# The Mitochondrial Theory of *g* Is Incompatible with Genetic Evidence and Does Not Explain Statistical Phenomena

**DOI:** 10.3390/jintelligence8030027

**Published:** 2020-06-28

**Authors:** Péter Przemyslaw Ujma, Kristof Kovacs

**Affiliations:** 1 Institute of Behavioural Sciences, Semmelweis University, 1089 Budapest, Hungary; ujma.peter@med.semmelweis-univ.hu; 2 National Institute of Clinical Neuroscience, 1145 Budapest, Hungary; 3 Institute of Psychology, ELTE Eotvos Lorand University, 1064 Budapest, Hungary

In two recent reviews (Geary 2018, 2019), Geary attributed a substantial role in generating individual differences in the general factor of intelligence, *g*, to mitochondrial functioning. While understanding the appeal of reducing a complex psychological phenomenon to an elementary biological cause and providing a new lease to Spearman’s theory of *g* as mental energy, we find the evidence supporting the theory to be rough-and-ready, indirect, or even contradictory. In particular, the theory lacks specificity in describing the causal path from mitochondria to *g* in two respects: (1) it would imply that genetic effects on *g* would exert their effect on mitochondria, which is at odds with current genetic evidence; (2) if *g* reflects variation in mitochondrial functioning and thus differences in *g* loadings necessarily indicate differences in the extent to which performance on a test depends on mitochondrial functioning, then the theory fails to account for why the effect of mitochondrial functioning on performance is greater in tests that have higher across-domain correlations.

First, the theory is contradicted by genetic studies of *g*. Cognitive ability is strongly heritable: based on quantitative genetic studies, in childhood around 50%, and in adulthood up to 80% of individual differences in cognitive ability or IQ scores can be ascribed to genetic differences (Plomin and Deary 2015; Polderman et al. 2015). Most studies use simple sum scores or first unrotated principal components of multiple cognitive tests as the dependent variable in quantitative genetic studies. However, when cognitive ability is decomposed to its hierarchical factor structure, *g* usually turns out to be even more heritable, while in less general abilities, genetic factors play a progressively weaker and environmental variables a progressively stronger role (Shikishima et al. 2009; Panizzon et al. 2014).

Quantitative genetic studies remain agnostic about the nature of the genetic determinants of a trait. However, recent large-scale genome-wide association studies (GWASs) revealed a large number of single nucleotide polymorphisms (SNPs) associated with cognitive ability (Lam et al. 2017; Savage et al. 2017; Trampush et al. 2017; Zabaneh et al. 2017; Davies et al. 2018; Hill et al. 2018). Even larger studies investigating the genetic correlates of educational attainment (Rietveld et al. 2013; Okbay et al. 2016; Lee et al. 2018) also found genetic variants which predicted cognitive ability. GWAS-derived polygenic scores currently predict up to 10% of cognitive performance (Lee et al. 2018; Allegrini et al. 2019). Within-family studies indicate that this effect size may be inflated due to population stratification, but a substantial proportion is still retained (Selzam et al. 2019). The functional interpretation of these genetic variants is not simple, because (1) many SNP hits are in intergenic regions with an unknown function, and (2) SNP hits are not necessarily causal for *g*; it is very likely that they are merely in linkage disequilibrium with truly functional variants in neighboring genomic regions. Still, multiple attempts have been made to at least approximately interpret the biological function of *g*-associated SNPs. These studies have unequivocally shown that *g*-associated genetic variants are primarily expressed in the brain, in specific brain regions and specific cell types, and they are implicated in very specific cellular functions, none of which concern mitochondria (Lam et al. 2017; Sniekers et al. 2017; Davies et al. 2018; Hill et al. 2018; Lee et al. 2018; Savage et al. 2018; Coleman et al. 2019). These findings are summarized in Table 1.

The current evidence suggests that the known genetic variants associated with individual differences in *g* affect specific areas of the brain, more specifically the frontal and anterior cingulate cortex, the cerebellum and certain subcortical structures. They seem to be expressed in specific cell types, and their functional role seems to be concentrated in neurogenesis, neuronal development and synaptic functions. This is in accordance with the watershed model that proposes that the causal effect of genotypes on intelligence as an observed phenotype is exerted through intermediate endophenotypes (Kievit et al. 2016).

In our view, these results are incompatible with individual differences in mitochondrial functioning playing a major role in creating individual differences in *g*. Mitochondria are present in all cells in all human tissues. If differences in mitochondrial function were to underlie individual differences in g, then *g*-associated genetic variants would not be expressed in specific tissues and cell types only. This would especially be the case if—as the hypothesis put forward by Geary (Geary 2018, 2019) suggests—the correlation between *g* and physical health (Calvin et al. 2011; Deary et al. 2019) exists because the same differences in mitochondrial functioning that create higher *g* in the central nervous system result in better physical health and greater longevity through their effects in other tissues. In our view, the functional role of *g*-associated SNPs is more consistent with the hypothesis that a large number of diverse, minor tissue- and cell-specific differences in the nervous system underlie *g*.

Second, since reflective latent variable models require a realistic ontology (Borsboom et al. 2003), if *g* in fact represents mitochondrial functioning, then in reflective models, *g* loadings must represent the extent of mitochondrial involvement. Since the general factor is a simple algebraic consequence of the positive manifold (Krijnen 2004), “it is always important to remember that it is the positive manifold, not *g* as such, that needs explanation” (Mackintosh 2011, p. 165). Therefore, translating *g* loadings in terms of the positive manifold itself, this means that the tests that correlate most strongly with other tests that have different content are the ones in which variation in performance depends most on mitochondrial functioning—according to Geary’s theory.

Hence, the theory should provide hypotheses regarding why such differences between *g* loadings in different tests depend on the relative involvement of mitochondrial function or energy. For instance, it is generally found that the more complex a task, the higher its *g* loading. However, complexity is not identical to difficulty. There are a number of manipulations that are able to increase *g* loadings, and the theory should be able to account for these. Why is mitochondrial functioning more relevant for backward digit span than for forward digit span? For odd-one-out reaction time than for simple reaction time?

Additionally, the factor Gf is found to be identical or near-identical to *g* (Gustafsson 1984; Kan et al. 2011), pointing to the centrality of fluid/inductive reasoning in *g*. Why does an inductive reasoning task, such as the completion of a number series or an incomplete matrix, require much more energy than a difficult short term memory task or a speed test, which requires one to work as fast as one can?

Even if the ultimate cause of differences in *g* loadings is mitochondrial functioning, a proximate psychological or physiological mechanism is needed that mediates this effect. Simply presuming that the stronger *g* loading is the result of such tasks’ higher “energy requirement”, without further explanation, would be tautological. If mitochondrial energy is more important for certain tasks and specific ability factors that are also most strongly related to *g,* then it should be explained why that is the case.

For the above reasons, our view is that individual differences in mitochondrial functioning probably do not underlie individual differences in *g*. The quest for the equivalent of psychological *g*, the common cause for the covariance in the performance on diverse cognitive tests, is still ongoing. In the meantime, theories and models that actually explain such covariance without assuming a common cause in the first place (Kovacs and Conway 2016; Savi et al. 2019; Van Der Maas et al. 2006) should probably also be considered.

## Figures and Tables

**Table 1:** Selected studies about the biological function of *g*-associated genetic variants. The last three columns highlight the organs, organ regions (typically brain regions) and cell types in which the genes mapped to *g*-associated single nucleotide polymorphisms (SNPs) were significantly enriched. We note that most of these studies used genetic data from multiple overlapping cohorts, hence cannot be considered independent.

Study	Data Source	N	Organ	Region	Cell Type or Function
Lam et al. 2017	Multiple cohorts also used in the Sniekers et al. 2017; Trampush et al. 2017; Okbay et al. 2016 GWASs.	107,207	Brain, pituitary	Cerebellar hemisphere, cerebellum, frontal cortex, cortex, anterior cingulate, nucleus accumbens, caudate nucleus, hypothalamus, hippocampus, putamen, amygdala	Neuron, neuron projection, neurogenesis, synapses, dendrites, synapse organization
Savage et al. 2018	UK Biobank, COGENT consortium and 12 other sources	269,867	Brain	Amygdala, anterior cingulate cortex, caudate nucleus, cerebellar hemisphere, cerebellum, cortex, frontal cortex, hippocampus, hypothalamus, nucleus accumbens, putamen	Medium spiny neuron, pyramidal (somatosensory, hippocampal CA1)
Davies et al. 2018	CHARGE and COGENT consortia, UK Biobank	300,486	Brain, pituitary	Cerebellum, cerebellar hemisphere, cortex, frontal cortex, hippocampus, nucleus accumbens, hypothalamus, amygdala, caudate nucleus, putamen, substantia nigra, pituitary	Neurogenesis, regulation of nervous system development, neuron projection, nervous system development, neuron differentiation, regulation of cell development, dendrites
Hill et al. 2018	Meta-analysis of the Sniekers et al. 2017; Okbay et al. 2016 GWASs, UK Biobank	248,482	Brain, pituitary	Cerebellar hemisphere, cerebellum, frontal cortex, cortex, anterior cingulate, nucleus accumbens, hippocampus, amygdala, hypothalamus, caudate nucleus, putamen, substantia nigra	Neurogenesis, nervous system development, cell development, neuron projection, CNS neuron differentiation, synapse, neuron differentiation, oligodendrocyte differentiation
Coleman et al. 2019	Meta-analysis of the Zabaneh et al. 2017; Sniekers et al. 2017 GWASs	87,740	Brain, pituitary	Frontal cortex	Pyramidal (somatosensory, hippocampal CA1), medium spiny neuron, embryonic GABAergic neuron, serotonergic neuron
